# Mimicking a submucosal tumor: endoscopic diagnosis and treatment of multiple mucosa-encapsulated fecaliths in the rectum

**DOI:** 10.1055/a-2928-6062

**Published:** 2026-09-08

**Authors:** Qiu-Jian Qiao, Wei He, Ying Zuo, Cheng Liu, Shan-Shan Li, Chang-Jiang Hu, Chao-Qiang Fan

**Affiliations:** 1 Department of Gastroenterology 105785 The Second Affiliated Hospital of Army Medical UniversityChongqingChongqingChina

**Fig. 1:**
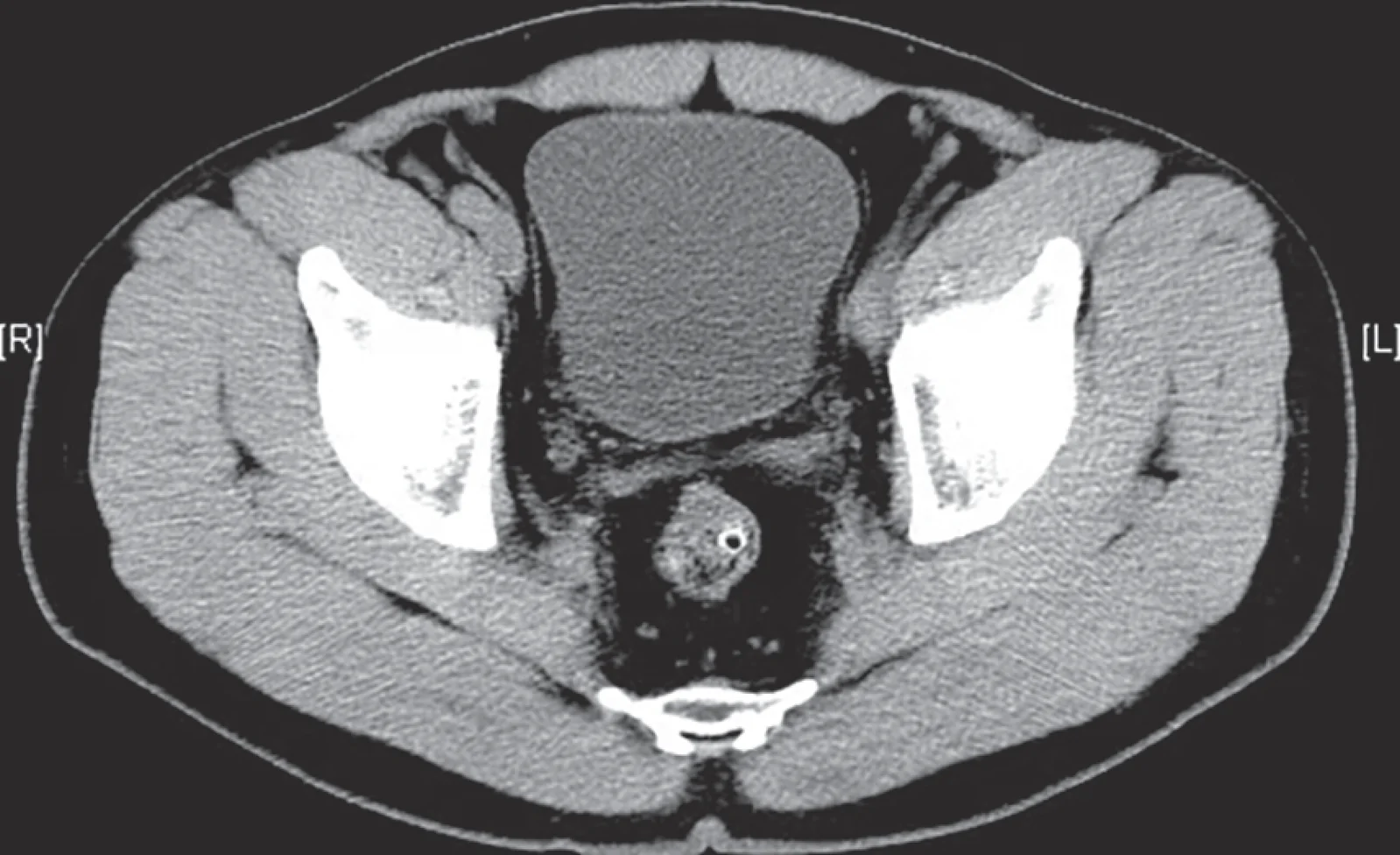
A CT scan revealed a well-defined, round/ovoid, focally calcified focus within the diverticulum, which displayed high attenuation (HU>100) and no contrast enhancement, characteristic of a fecalith.

**Fig. 2:**
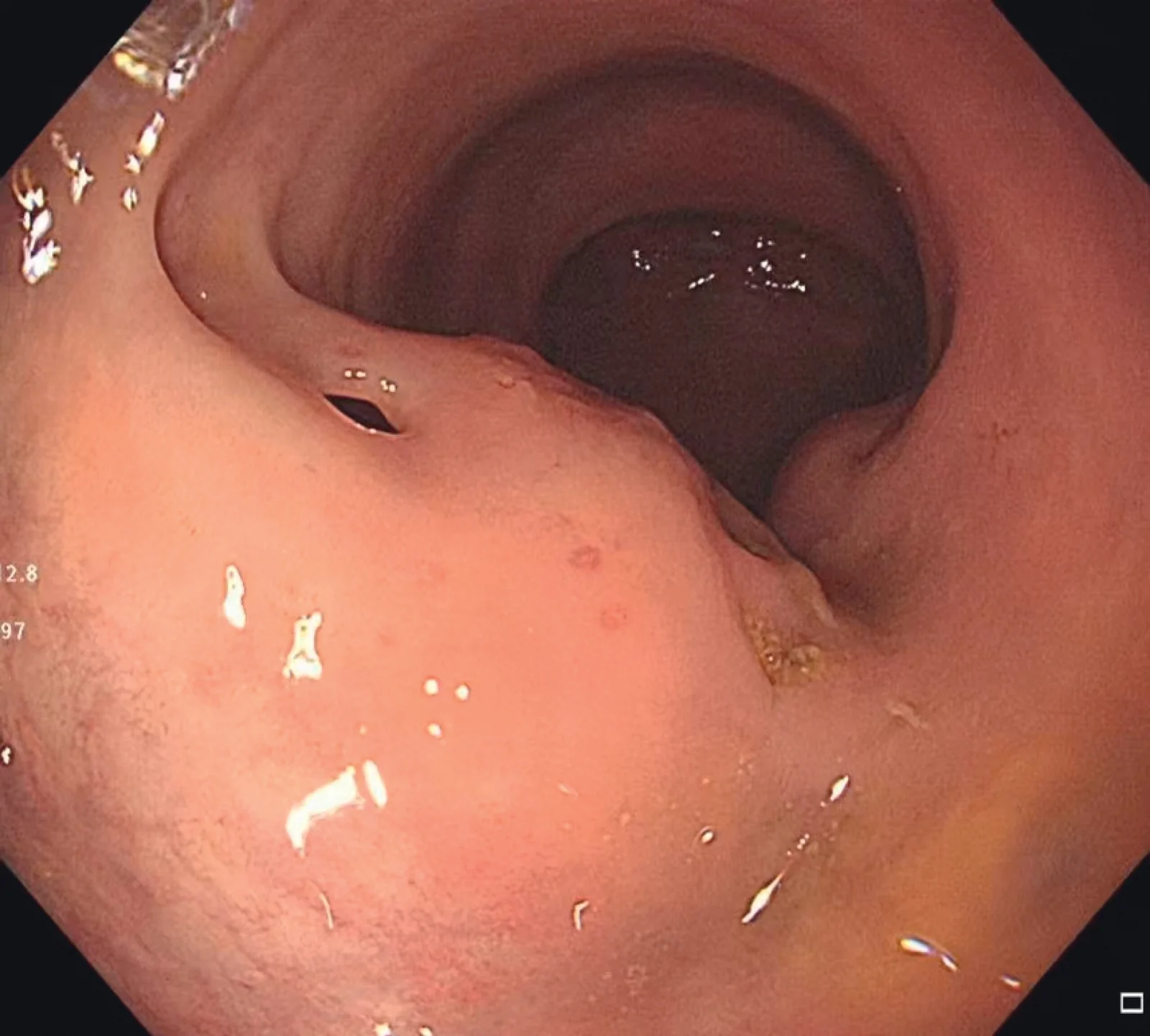
Colonoscopy identified a smooth, firm, and minimally mobile submucosal elevation in the rectum, featuring two 2-mm-diameter openings on the surface.

**Fig. 3:**
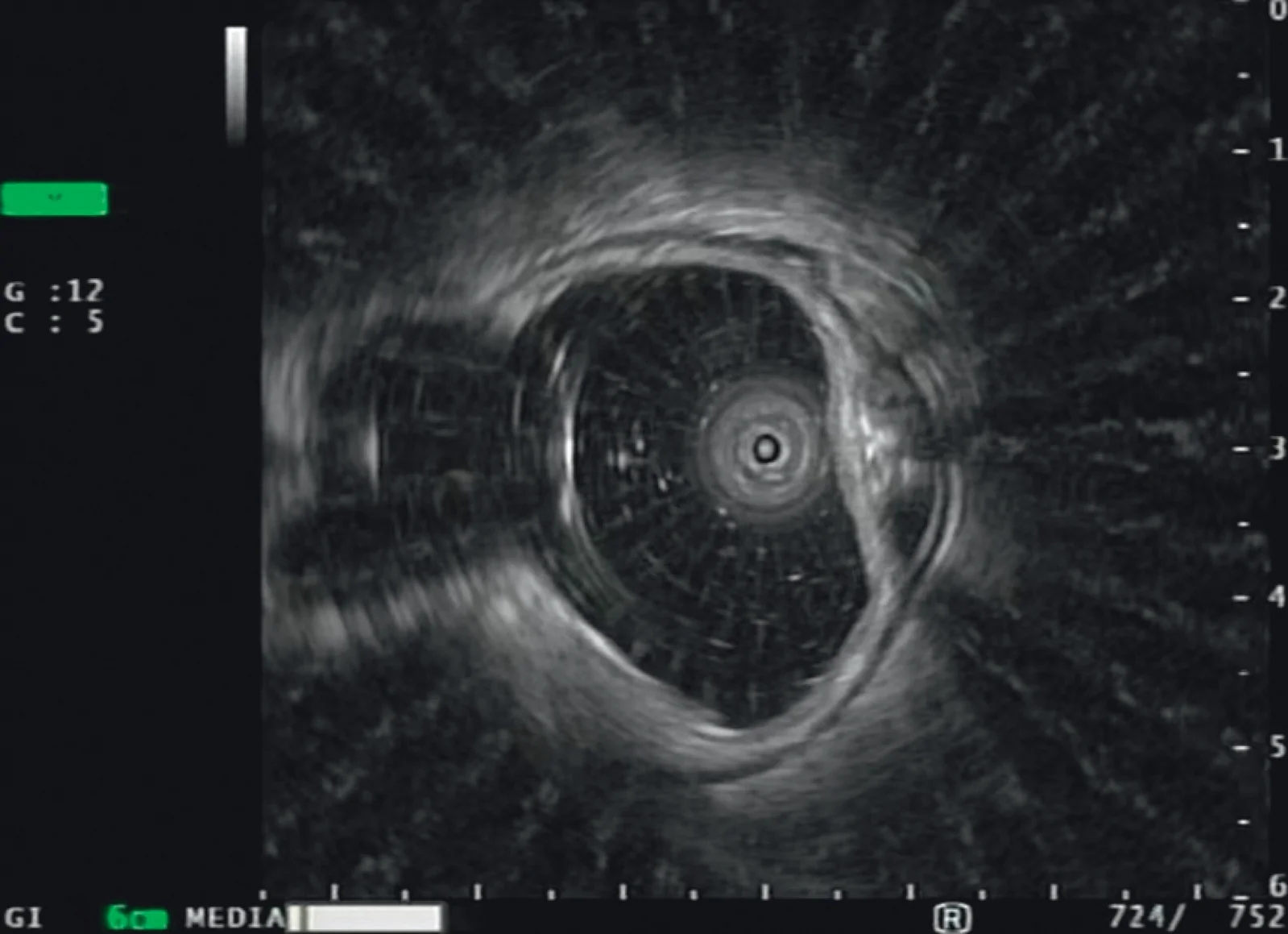
Endoscopic ultrasonography (EUS) revealed a hyperechoic focus with a posterior acoustic shadow within a rectal diverticulum, distinguishing it from a submucosal tumor.

Rectal diverticulum with a fecalith is an exceptionally rare clinical phenomenon that is frequently misdiagnosed as a rectal submucosal tumor. 1​ A 50-year-old man with a 3-year history of chronic constipation was admitted with a 2-month history of altered bowel habits, manifesting as dyschezia and altered stool consistency, without hematochezia, mucus in stool, or abdominal pain. Abdominal computed tomography ( Fig. 1 ) revealed rectal wall thickening, with no evidence of peritoneal effusion or perirectal inflammation. Colonoscopy ( **Fig. 2** ) identified a smooth, firm, and minimally mobile submucosal elevation in the rectum, featuring two openings of 2 mm in diameter on its surface. Endoscopic ultrasonography (EUS; Fig. 3 ) revealed a hyperechoic focus with a posterior acoustic shadow within a rectal diverticulum, distinguishing it from a submucosal tumor.

**Fig. 4:**
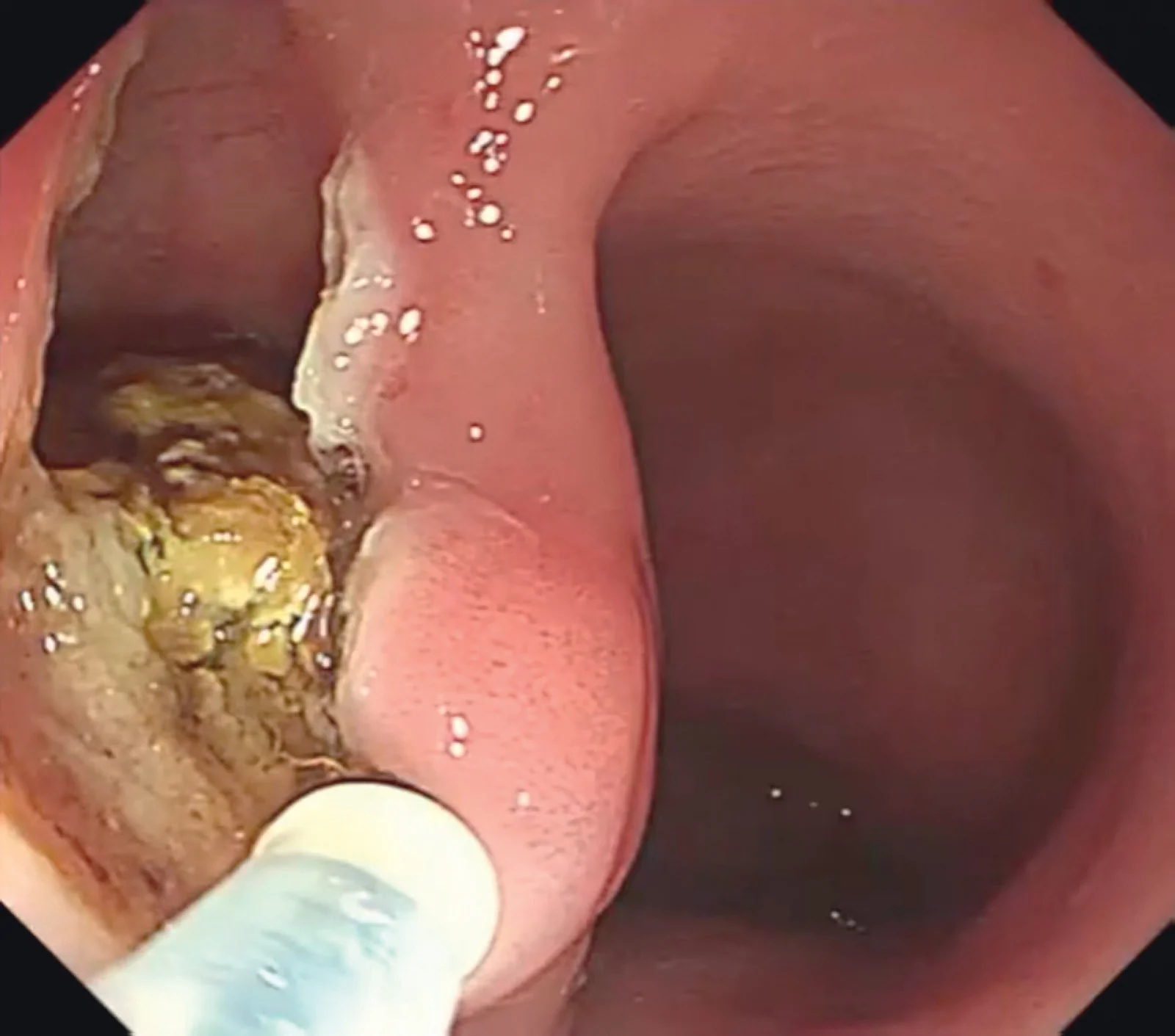
An IT knife was used to incise the overlying mucosa, creating a “window” .

**Fig 5:**
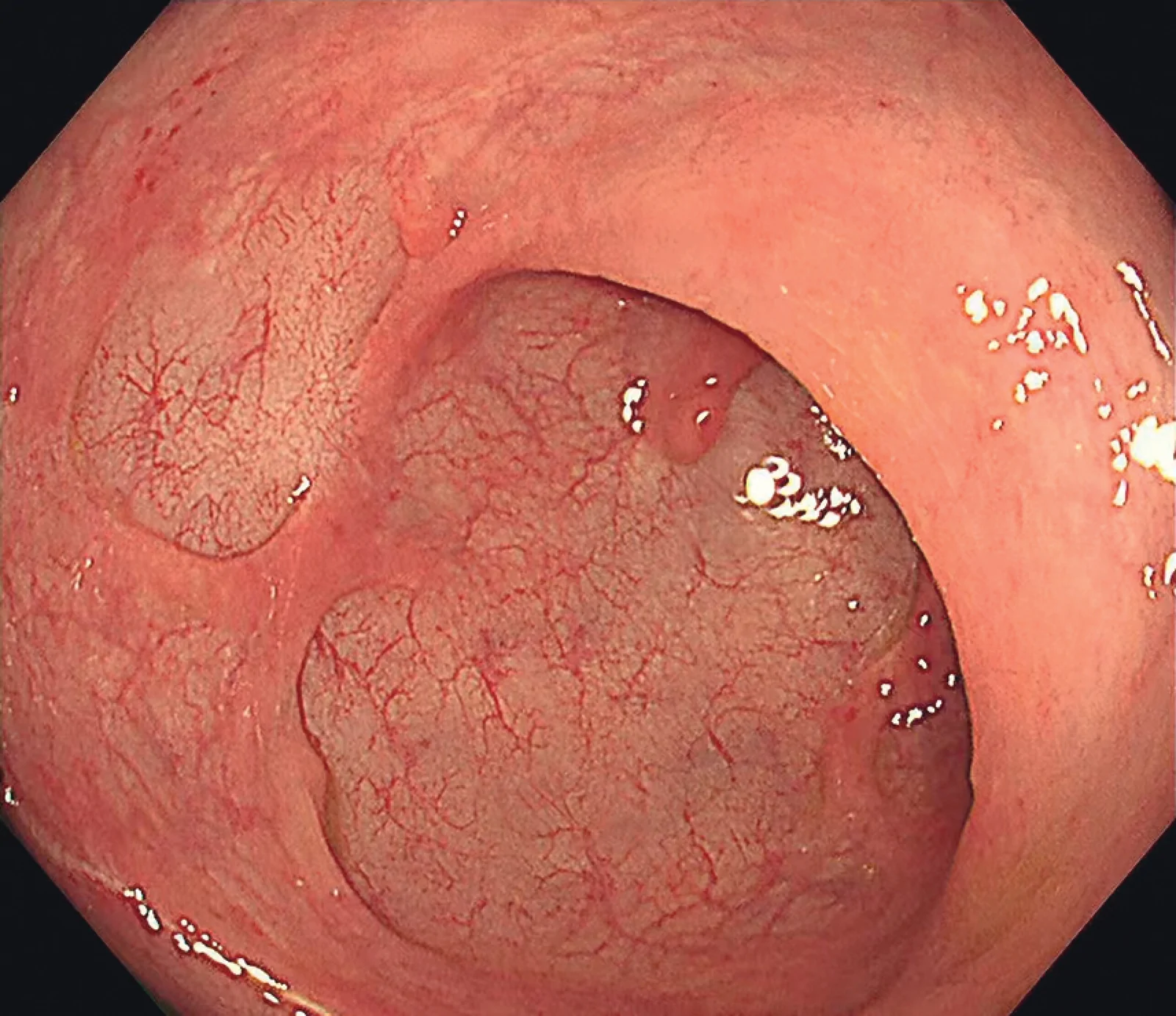
A colonoscopy performed 1 year postoperatively confirmed that the wound had healed well, with no residual pseudocavity or recurrence.

After obtaining comprehensive informed consent regarding the risks of bleeding and perforation, endoscopic diverticular fenestration was performed ( Video 1 ). An IT knife was used to incise the overlying mucosa, creating a “window” through which two fecaliths, measuring 3 cm and 1.2 cm, respectively, were identified and removed ( Fig. 4 ). The patient was kept on a 24-hour fast and discharged on the third postoperative day without complications. His preoperative symptoms (dyschezia and altered bowel habits) completely resolved after the procedure. A colonoscopy performed 1 year postoperatively confirmed that the wound had healed well, with no residual pseudocavity or recurrence ( Fig. 5 ).

**Video 1** Endoscopic diverticular fenestration was used to treat mucosa-encapsulated fecaliths in the rectum. Video URI:


EUS is a valuable tool for differential diagnosis in such cases, 2​ ​3​ and endoscopic diverticular fenestration represents a safe and effective minimally invasive treatment option.

Endoscopy_UCTN_Code_CCL_1AD_2AI
